# Measuring Rolling Friction at the Nanoscale

**DOI:** 10.1021/acs.langmuir.3c03499

**Published:** 2024-03-18

**Authors:** Simon Scherrer, Shivaprakash N. Ramakrishna, Vincent Niggel, Nicholas D. Spencer, Lucio Isa

**Affiliations:** Department of Materials, ETH Zürich, Zürich 8093, Switzerland

## Abstract

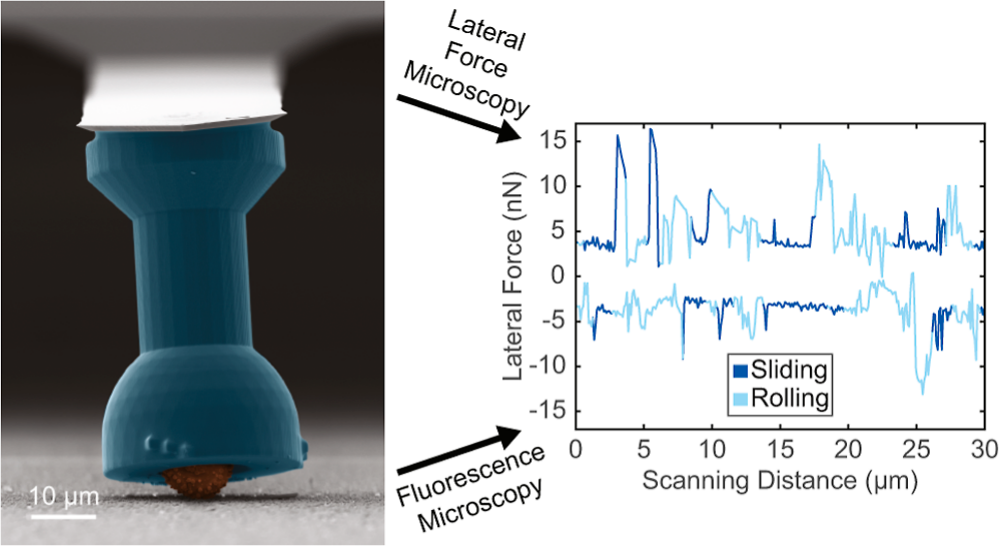

Colloidal probe microscopy, a technique whereby a microparticle is affixed at the end of an atomic force microscopy (AFM) cantilever, plays a pivotal role in enabling the measurement of friction at the nanoscale and is of high relevance for applications and fundamental studies alike. However, in conventional experiments, the probe particle is immobilized onto the cantilever, thereby restricting its relative motion against a countersurface to pure sliding. Nonetheless, under many conditions of interest, such as during the processing of particle-based materials, particles are free to roll and slide past each other, calling for the development of techniques capable of measuring rolling friction alongside sliding friction. Here, we present a new methodology to measure lateral forces during rolling contacts based on the adaptation of colloidal probe microscopy. Using two-photon polymerization direct laser writing, we microfabricate holders that can capture microparticles, but allow for their free rotation. Once attached to an AFM cantilever, upon lateral scanning, the holders enable both sliding and rolling contacts between the captured particles and the substrate, depending on the interactions, while simultaneously giving access to normal and lateral force signals. Crucially, by producing particles with optically heterogeneous surfaces, we can accurately detect the presence of rotation during scanning. After introducing the workflow for the fabrication and use of the probes, we provide details on their calibration, investigate the effect of the materials used to fabricate them, and report data on rolling friction as a function of the surface roughness of the probe particles. We firmly believe that our methodology opens up new avenues for the characterization of rolling contacts at the nanoscale, aimed, for instance, at engineering particle surface properties and characterizing functional coatings in terms of their rolling friction.

## 1 Introduction

Colloidal materials, i.e., systems comprising micro- and nanoscale objects suspended in a fluid, are ubiquitous in our daily lives and in a broad range of technological applications. Their properties are rooted in the profound impact of surface forces^1^ and nanometer–scale interactions,^2,3^ which, for instance, affect the colloidal stability of suspensions^4^ and the aggregation behavior of particles.^5^ Atomic force microscopy (AFM) has emerged through the decades as a key analytical technique for the precise measurement of colloidal interactions at the nanometer level,^6^ where a cantilever, typically made of silicon or silicon nitride, with a sharp tip is used as a probe.^7^ However, the direct measurement of forces between individual particles is also possible if, in place of a sharp tip, a spherical particle of interest, generally in the range of 1–100 μm, is permanently attached to the end of an AFM cantilever by means of glue or a sintering process to obtain so-called colloidal probes.^8,9^ The interactions between the probe and the countersurface, which can be a second particle or a planar substrate, are then measured by recording the deflection of the cantilever as a function of the vertical displacement of the AFM Z-scanner. After calibration of the cantilever’s spring constant and the deflection sensitivity, one can extract the force as a function of distance, known as a force-vs-distance curve (or *F*–*D* curve).^10^

In addition to normal force measurements obtained from *F*–*D* curves, AFM is also extensively used to measure nanoscale lateral forces, in a mode generally known as lateral force microscopy (LFM), thus providing access to friction forces between the colloidal probe and the substrate. In LFM, after calibrating its torsional spring constant, a cantilever is moved laterally over a certain distance on the sample surface while keeping the normal force constant, and the cantilever’s lateral deflection under sliding is recorded to quantify friction at the contact. In the recent past, LFM has emerged as the cornerstone of the nanotribology community, providing a crucial avenue for exploring the molecular-level contact between particles and substrates in relative sliding motion and shedding light on the underlying mechanisms behind lubrication and wear.^11−13^ Colloidal probe LFM furthermore enables investigations of nanoscale adhesion, friction, and contact mechanics to elucidate many fundamental questions in tribology and rheology^14^ as well as in colloidal and interface science.^15,16^ The technique has also gained significant demand in various industries where understanding the forces between contacting surfaces is crucial, particularly in microelectromechanical systems (MEMS), surface coatings, printing, powders, and pastes, just to name a few.^17,18^

Despite its conceptual simplicity and versatility, colloidal probe LFM still suffers from some drawbacks. Currently, many separate colloidal probes must be prepared to acquire statistically significant data sets, and limitations exist in the type of particles that can be attached.^19^ These obstacles can be, at least partially, overcome by techniques that allow for in situ immobilization of particles, e.g., via sophisticated fluidic systems^20,21^ or cantilever functionalization.^22^ However, and most importantly as the motivation for this work, fixing a particle to the cantilever only allows for sliding against the countersurface and prevents any other mode of relative motion. Nonetheless, in almost all technologically relevant processing of colloidal systems, particles are freely suspended in a medium or are present in a powder form. Interacting particles are free to translate and rotate relative to each other, and both sliding and rolling frictions are present and important. With conventional colloidal probes, any rotation of the particle is restrained, and measuring the rolling friction is therefore impossible. Moreover, any accurate measurement of rolling friction also requires an accurate measurement of relative rotation, which is not accessible with a conventional AFM.

To this end, several approaches have been proposed. Sitti et al. initiated the idea of pushing microparticles with a conventional, sharp AFM tip to study rolling and spinning in addition to pure sliding.^23,24^ They concluded that upon application of a tangential force, particles were undergoing a combination of sliding and rolling motion. A direct detection of the distinction between sliding and rolling during manipulation was proposed by imaging partially bleached, fluorescent micron-sized particles with an optical microscope by Schiwek et al.^25^ or by imaging them in an SEM vacuum chamber.^26^ In the case of nanoparticles, the type of motion could only be verified indirectly by comparing theoretical calculations to experimental results.^27^ The rolling resistance of selected microparticles was also investigated by compressing particle chains with an AFM cantilever^28^ and with optical tweezers^29^ or by rolling particles between two flat planes.^30^ Experiments have been accompanied by theoretical studies, where, for instance, by examining the stability of agglomerates of micron-sized particles, Dominik et al. calculated the resistance to rolling based on the description of contact forces by Johnson, Kendall, and Roberts,^31^ and adhesion hysteresis.^32^ Dynamic models developed by Korayem and Zakeri focused on the materials properties,^33^ providing valuable inputs to back experimental results up.

The literature provides overwhelming evidence that nano- and microparticles tend to roll rather than slide when tangential forces are applied. Nevertheless, colloidal probe studies, which exclusively measure sliding friction, have dominated the field of nanotribology due to experimental difficulties arising from measuring the forces on a free particle. The approaches described above, while providing valuable insights, cannot fully capture the dynamic measurement of rolling frictional forces for free particles suspended in a liquid, while simultaneously quantifying the type of relative motion at contact. A new experimental method is therefore required, combining lateral and normal force acquisition of free particles with direct real-time visualization of the contact.

**Figure 1:**
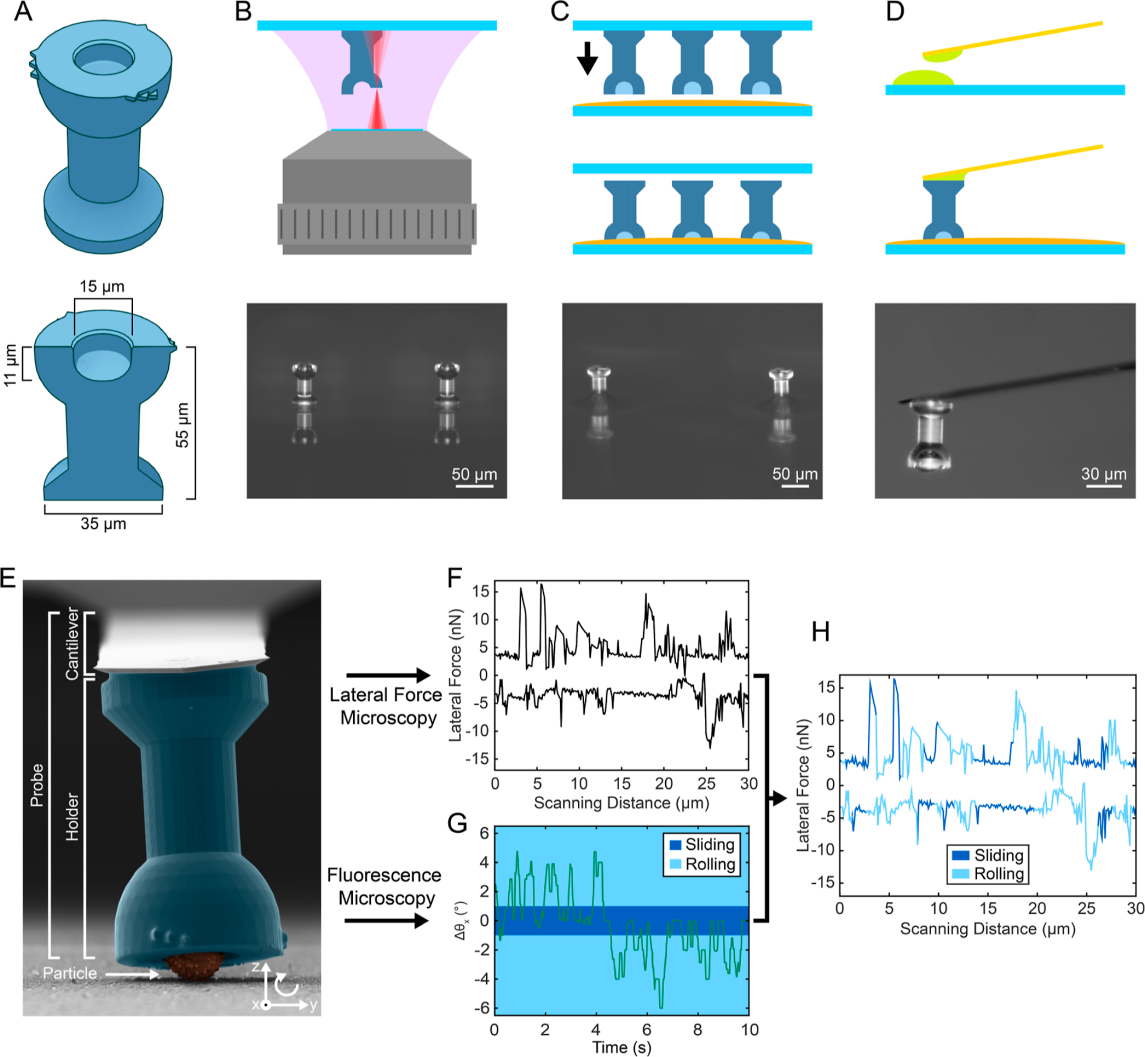
Fabrication of the probe and experimental procedure. (A) Design and dimensions of the holder used for microparticle capture. (B) Schematic of 3D printing of the holders by 2PP-DLW and optical micrographs of the prints. (C) Transfer of the holders onto a sacrificial glucose layer on top of a glass slide and optical micrograph after transfer. (D) Probe assembly by gluing a single holder onto a tipless AFM cantilever with UV glue and optical micrograph of the probe. (E) Representative SEM image (false-colored) of a particle (red), captured by the holder (blue). (F) Friction loop obtained by performing standard lateral force microscopy. (G) Angular displacement of the probe particle in the scanning direction as a function of time. Rolling is detected if the angular displacement exceeds 2° between consecutive frames. (H) Friction loop, color-coded to visualize and separate the rolling and sliding motion of the particle.

In this paper, we introduce a novel approach for quantifying the rolling friction of microparticles. Our method offers the unique combination of free particle rotation during scanning (monitored via fluorescence microscopy) with the experimental versatility of conventional colloidal probe LFM. This is realized by fabricating a custom particle holder and fixing it to the end of a tipless AFM cantilever to construct the probe, as seen in Figure 1A–E. More details on the probe assembly and working principle can be found in the Experimental Section and the Results and Discussion. In the present study, we use raspberry (RB) particles^34,35^ with fluorescent markers that allow for rotational tracking. The used RB particles comprise a 12 μm silica microparticle, decorated with silica nanoparticles, ranging in size from 100 to 500 nm. The nanoparticles are permanently attached and form the primary topographical features, which we refer to as asperities in this paper, as described in Figure S1 and the Experimental Section, where we also describe the fabrication and calibration of the probe, the synthesis of the particles and substrates used in this study, and the experimental workflow we established to investigate rolling friction at the nanoscale. In the Results and Discussion, we go into the details of how our method can expand the possibilities of colloidal probe LFM by examining how the motion of a particle depends on both the experimental conditions and the particle–substrate contact. We in particular show that said contact can involve sliding, rolling, or both. Additionally, side-by-side comparisons of friction measured for a free and fixed particle are possible. The prepared probes can be reused several times, enabling experiments to be performed with many particles.

## 2 Experimental Section

If not stated otherwise, all chemicals were used as provided by the supplier. Ethanol (EtOH, 96%), propylene glycol monomethyl ether acetate (PGMEA, 99.5%), 2-propanol (IPA, 99.5%), toluene (99.5%), glucose (99.5%), ammonia (25% in water), hydrogen peroxide solution (30% in water), poly(diallyldimethylammonium chloride) solution (PolyDADMAC, 20% w/v in water, 400–500 kDa), tetraethyl orthosilicate (TEOS, 99%), and polyethylenimine (PEI, *M*_n_ = 10,000) were purchased from Sigma-Aldrich (Switzerland).

### 2.1 Probe Fabrication

The holder was designed in AutoCAD 2022 and exported as a .stl-file. We have saved the file in a data repository to enable other users to print their own (see Data Availability in the back). The final dimensions and shape used for the presented experiments are listed in Figure 1A. A fused silica substrate (Multi-Dill, NanoScribe GmbH, Germany) was cleaned with the standard procedure from NanoScribe (EtOH rinse, plasma-treated for 20 s using normal pressure plasma in ambient air with a Piezobrush PZ2 (relyon plasma GmbH, Germany)). A commercial two-photon polymerization direct laser writing (2PP DLW) setup (Photonic Professional GT2, NanoScribe GmbH, Germany) with a 63× NA = 1.5 objective and commercial Dip-in resins (IP-Dip and IP-S, NanoScribe GmbH, Germany) were used. A set of holders (20–30) was printed using the standard printing recipe for IP-Dip, as illustrated in Figure 1B. The prints were developed with the standard procedure (20 min PGMEA, 5 min IPA, dried with nitrogen) and postcured with UV light (365 nm) for 1 h. A solution of glucose in Milli-Q water (40% w/v) was prepared and spin-coated (Laurell Technologies Corp., USA) at 4000 rpm for 15 s on a microscope slide (Thermo Fisher Scientific, Switzerland) that had been previously plasma-treated for 20 s using normal pressure plasma in ambient air (Piezobrush PZ2, relyon plasma GmbH, Germany). As shown in Figure 1C, the two substrates were brought into direct contact without pressing them together. When separated, the prints stuck to the glucose and were released from the original substrate. The integrity of the prints was confirmed by using optical microscopy (BX41, Olympus, Switzerland). Once the print was transferred to the substrate with glucose, the prints can be stored for months. The fabricated holders were attached to tipless AFM cantilevers (HQ/CSC38/tipless/Cr–Au and HQ/NSC35/tipless/Al BS, MikroMasch, Bulgaria). First, the normal and torsional spring constant (*K*) and quality factor were measured in air with an AFM (Nanowizard III, JPK, Germany). To optimize attachment, the cantilever and the substrate with prints were UV–ozone cleaned (185 and 254 nm LED, 15 mW·cm^–2^, Ossila Ltd., UK) for 10 min. The cleaned cantilever was mounted on AFM (Dimension Icon, Bruker, USA), and a small amount of UV-adhesive (Norland Optical Adhesive 81, USA) was added to a glass slide. Small drops were created by spreading the glue using a micropipette tip. Next, the edge of a small drop was touched briefly with the cantilever by operating the AFM in contact mode without scanning to restrict any horizontal displacement (see Figures 1D and S2). The end of the cantilever was then centered on the base of a holder and brought into contact at a low applied normal force (around 10 nN). The UV-adhesive was cured by using a UV flashlight (GEM10 UV, 3000 mW at 365 nm, Nitecore, Germany) for 1 min at a distance of 3–4 cm. The glucose layer was softened by increasing the local humidity, i.e., by simply placing a piece of wet paper towel next to the stage, and the holder was released from the substrate upon retracting the cantilever. Glucose residues were removed by submerging the cantilever in Milli-Q water for 10 min. Alignment and integrity of the holder were confirmed by using optical microscopy (BX41, Olympus, Switzerland), scanning electron microscopy (Gemini Leo-1530, Germany), and AFM (Dimension Icon, Bruker, USA).

### 2.2 Raspberry Particles with Fluorescent Markers

The raspberry (RB) particles were synthesized based on a previously reported heterogeneous aggregation method^34,35^ and adapted for larger silica microparticles of 12 μm diameter (microParticles GmbH, Germany). The surfaces of the particles were cleaned by adding 0.2 mL of silica particles (5% w/v) to a mixture of 1 mL of ammonia (25% in water) with 1 mL of H_2_O_2_ (30% in water) and 0.8 mL of Milli-Q water that had been heated to 70 °C. The slurry was stirred for 10 min before washing the particles in Milli-Q water by repeated centrifugation. The surface charge of the particles was inverted by suspending them in 10 mL of an aqueous solution of PolyDADMAC at a concentration of 0.025% w/v and stirring for 1 h. The particles were washed by repeated centrifugation in Milli-Q water. The nanoparticles were attached by mixing the positively charged microparticles in 10 mL of Milli-Q water with fluorescent polystyrene particles (100 nm/0.875 mL of a 0.005% w/v suspension from Invitrogen, USA, 200 nm/2.2 μL of a 2% w/v suspension from Invitrogen, USA, 300 nm/1.76 μL of a 2.5% w/v suspension from microParticles GmbH, Germany, 500 nm/4.6 μL of a 2.5% w/v suspension from microParticles GmbH, Germany) under stirring for 20 min followed by silica nanoparticles (100 nm/20 μL of a 1% w/v suspension from nanoComposix, USA, 200 nm/20 μL of a 2% w/v suspension from nanoComposix, USA, 300 nm/40 μL of a 1% w/v suspension from nanoComposix, USA, 400 nm/4 μL of a 5% w/v suspension from microParticles GmbH, Germany, 500 nm/24.2 μL of a 5% w/v suspension from microParticles GmbH, Germany) and stirring for another 80 min. The raspberry particles were synthesized using the same size of fluorescent polystyrene and silica nanoparticles, except for 400 nm silica nanoparticles, in which case 300 nm polystyrene nanoparticles were used as trackers. To separate the nanoparticles that were not attached, the RB particles were left to sediment and the supernatant was removed to concentrate the particle suspension into 1 mL. A silica layer was grown on the surface of the particles to bind the nanoparticles permanently to the microparticles and to have a consistent silica surface chemistry. 7.5 mL of EtOH and 1.3 mL of ammonia were added to the suspension. A solution of 5% v/v TEOS in EtOH was added in several steps. First, 0.25 mL was added at 2 mL/h before stirring for 30 min. This process was repeated once more before adding another 0.25 mL of the solution at 2 mL/h while sonicating the dispersion (SONOREX DIGIPLUS, Bandelin GmbH, Germany). Finally, the particles were cleaned by repeated centrifugation before they were stored in Milli-Q water. The particles were analyzed by means of scanning electron microscopy (Gemini Leo-1530, Germany) and AFM (Dimension Icon, Bruker, USA).

### 2.3 Rough Substrates

The substrates were prepared according to a protocol developed during previous work.^36^ Glass cover slides (18 × 18 mm, #1, Menzel-Gläser, Germany) were sonicated in toluene, IPA, EtOH, and Milli-Q water for 10 min each, followed by 20 min UV–ozone (185 and 254 nm LED, 15 mW·cm^–2^, Ossila Ltd., UK) cleaning. The substrates were positively charged by submersion in a solution of PEI (1 mg/mL) for 30 min while stirring, before rinsing them with Milli-Q water and drying them with a nitrogen jet. The substrates were submerged into aqueous dispersions of silica nanoparticles that adsorbed onto the substrates electrostatically. The coverage is a function of the concentration and the time that the substrates were exposed to the dispersion. For 100 and 200 nm silica nanoparticles, the substrates were submerged for 20 min into a 0.04 and 0.02% w/v dispersion, respectively. The samples were rinsed with Milli-Q water and exposed to a TEOS solution to mimic the surface of the raspberry particles. The substrates were added to a beaker containing 7.44 mL EtOH, 1.22 mL ammonia, 1 mL Milli-Q water, and 0.6 mL of 1% v/v ethanolic TEOS solution and stirred for 30 min. Finally, the substrates were rinsed with Milli-Q water and dried in a nitrogen jet. The substrates were analyzed with scanning electron microscopy (Gemini Leo-1530, Germany) and AFM (Dimension Icon, Bruker, USA).

### 2.4 Substrates with Roughness Steps

We fabricated substrates presenting steps in surface roughness in order to verify changes in contact mode along the same lateral displacement tracks. To this end, a rough substrate, comprising 200 nm silica nanoparticles attached to a glass substrate, was prepared according to the previous section. To recover the pristine, smooth glass slide on a section of the substrate, nanoparticles were removed by rubbing the surface with a soft plastic tip, resulting in a sharp transition from smooth to rough that was easily identified by optical microscopy. The substrate was then rinsed with EtOH and Milli-Q water before 20 min UV–ozone (185 and 254 nm LED, 15 mW·cm^–2^, Ossila Ltd., UK) cleaning to remove any residue.

### 2.5 Details on Rotational Particle Tracking

The tracking of the rotation of the optically anisotropic particles is based on a method reported by Niggel et al.^37^ First, the center of the particle is found in each frame of a time series of standard wide-field epi-fluorescence microscopy images (Axio Observer D1, 40× NA = 0.6 objective, Filter Set 09, Zeiss, Germany, 89 North Photofluor II, USA, Zyla 4.2, Andor, UK, acquisition rate: 25 frames per second, 2048 × 2048 pixels). Then, using image correlation, a reference image is compared with sequentially rotated images of a subsequent frame to find the most probable change in angular orientation. The instantaneous rotation of the particle can be extracted and synchronized with the friction force, as seen in Figure 1F–H. Alternatively, the cumulative rotation in one scan can be determined and compared to the theoretical rolling without slip of a particle of the same diameter over the same distance. The rotation can be displayed as a percentage compared to pure rolling without slip, as shown later in the article.

### 2.6 Calibration-Wedge Fabrication

The calibration wedges^38^ were designed in AutoCAD 2022 and exported as a .stl-file. They are 100 μm long with a slope of 10, 15, 20, and 25°. Their widths are 40, 45, 50, and 55 μm, respectively, to distinguish them. The length of the base is 40 μm and the top is 20 μm. A fused silica substrate (Multi-Dill, NanoScribe GmbH, Germany) was cleaned with the standard procedure from NanoScribe (EtOH rinse, plasma-treated for 20 s using normal pressure plasma in ambient air with a Piezobrush PZ2 (relyon plasma GmbH, Germany)). A commercial 2PP DLW setup (Photonic Professional GT2, NanoScribe GmbH, Germany) with a 63× NA = 1.5 objective and commercial Dip-in resin (IP-Dip, NanoScribe GmbH, Germany) was used to print all calibration wedges on a single substrate. The prints were developed with the standard procedure (20 min PGMEA, 5 min IPA, dried with nitrogen) and postcured with UV light (365 nm) for 1 h.

### 2.7 Test-Probe Fabrication

For the test-probe calibration method,^39^ the normal and torsional resonance frequency and quality factor of a UV–ozone-cleaned cantilever (HQ/CSC38/tipless/Cr–Au, MikroMasch, Bulgaria) were determined with an atomic force microscope (Nanowizard III, JPK, Germany) to calculate the normal and torsional spring constants.^40^ Glass particles (50–100 μm, Whitehouse Scientific, UK) were deposited on a microscope slide, dried with a nitrogen jet, and mounted on the AFM stage. The tip of the cantilever was dipped into a small drop of UV-curable adhesive (Norland Optical Adhesive 63, USA) and aligned with a particle. The cantilever was brought into contact with the particle and the adhesive was cured with UV light (GEM10 UV, 3000 mW at 365 nm, Nitecore, Germany) for 1 min at a distance of 3–4 cm. A clean and sharp edge of the silicon wafer was used as a vertical sidewall for the measurement of lateral deflection sensitivity.

## 3 Results and Discussion

### 3.1 Fabrication of the Holder

The key step of our methodology is the ability to realize AFM cantilevers that are capable of temporarily capturing individual particles and manipulating them, while allowing for the simultaneous measurement of lateral forces and the visualization of their motion without immobilization. To achieve this goal, we fabricate a microprinted concave particle holder and attach it to a commercially available tipless AFM cantilever, as described in the Experimental Section. Figure 1A–D shows a schematic representation of the individual fabrication steps as well as the corresponding optical-microscopy images. The main fabrication workflow consists of four steps. Initially, a design for the concave holder was developed. This includes a large contact area at the base, a long pillar to enhance the torsional arm, a hemispherical cavity for capturing a single particle, and focus indicators to confirm that the holder is not contacting the substrate below. All experiments in this study were performed with the holders displayed in Figure 1A, but the CAD model can be adapted to suit other requirements. Following the design, sets of 20–30 holders were fabricated in about 1 h with a 3D-printing technique based on two-photon polymerization direct laser writing (2PP DLW), as shown in Figure 1B. They were printed with the opening of the holder facing away from the substrate to allow for the complete removal of uncured resin during development. Subsequently, the entire printed holder array was transferred to a glucose-coated substrate, resulting in the inversion of the holder orientation relative to the substrate to which they are affixed, as seen in Figure 1C. The base of the holder, which was originally in contact with the substrate, was therefore facing away from the glass slide. Additionally, the adhesive strength of the glucose layer holding the prints on the substrate could be controlled via the local humidity, which was crucial for the next step. Finally, the probe was assembled by attaching a single holder to the end of a tipless AFM cantilever. Following the procedure outlined in Figure 1D, the cantilever end was first gently brought into contact with a small drop of UV-curable adhesive before being aligned with the base of a printed holder. After contact, the adhesive was cured, and the glucose layer was softened to release the holder from the transfer substrate. Additional details about the probe fabrication can be found in the Experimental Section.

### 3.2 Free-Colloidal Probe Concept

An integrated setup, combining an AFM with lateral-deflection signal readout and an inverted optical microscope for imaging, is required for the simultaneous measurement of friction and contact visualization. Moreover, measuring the orientation and angular displacements of a spherical particle necessitates optical anisotropy in the particle, which can be achieved either during synthesis^41−43^ or afterward.^25^ The 3D rotation of optically anisotropic particles can be tracked from full 3D confocal images,^35,44^ or, for particles whose translational motion is restricted to the *XY*-plane, by an alternative method that relies on 2D wide-field microscopy images and allows for faster acquisition rates and simpler imaging conditions (see Experimental Section).^37^ In our experiments, we choose the latter method and apply it to track optically anisotropic raspberry particles (see Figure S1) prepared by a previously reported method based on heterogeneous aggregation.^34,35^

A diluted suspension of these particles, dispersed in an aqueous buffer, was left to sediment onto a transparent substrate, which was prepared as detailed in the Experimental Section. The 3D-printed probe was mounted on the AFM head (Nanowizard III, JPK, Germany) and aligned by means of the laser and detector signal. In the next step, we centered the holder cavity above a particle, which was captured by carefully approaching the probe onto the substrate. The false-colored SEM image in Figure 1E illustrates the final experimental setup, with a particle captured by the probe. We then performed standard lateral-force-microscopy experiments to obtain friction loops by laterally displacing the particle orthogonally to the long cantilever axis, i.e., along the *y*-axis (see Figure 1F). Parameters such as applied normal force, scanning distance, and scan speed were simply set analogously to friction-force measurements with conventional, fixed colloidal probes. For all of the data presented here, the scan speed was fixed to 10 μm/s. We simultaneously imaged the contact region below the particle via fluorescence microscopy (Axio Observer D1, Zeiss, Germany) and extracted the instantaneous 3D rotation around the *x*-axis of the particle from the 2D images, as illustrated in Figure 1G (more details in the Experimental Section). The major axis of rotation is around the *x*-axis and is denoted as θ_*x*_, as illustrated in Figure 1E. While rotations around the other axes can also be detected, the overall angular displacement around the *x*-axis is much larger and is reported here. Finally, lateral-force and rotational dynamics measurements were combined, making it possible to resolve local correlations between friction and particle motion (Figure 1H).

### 3.3 Calibration of Friction Forces

**Figure 2:**
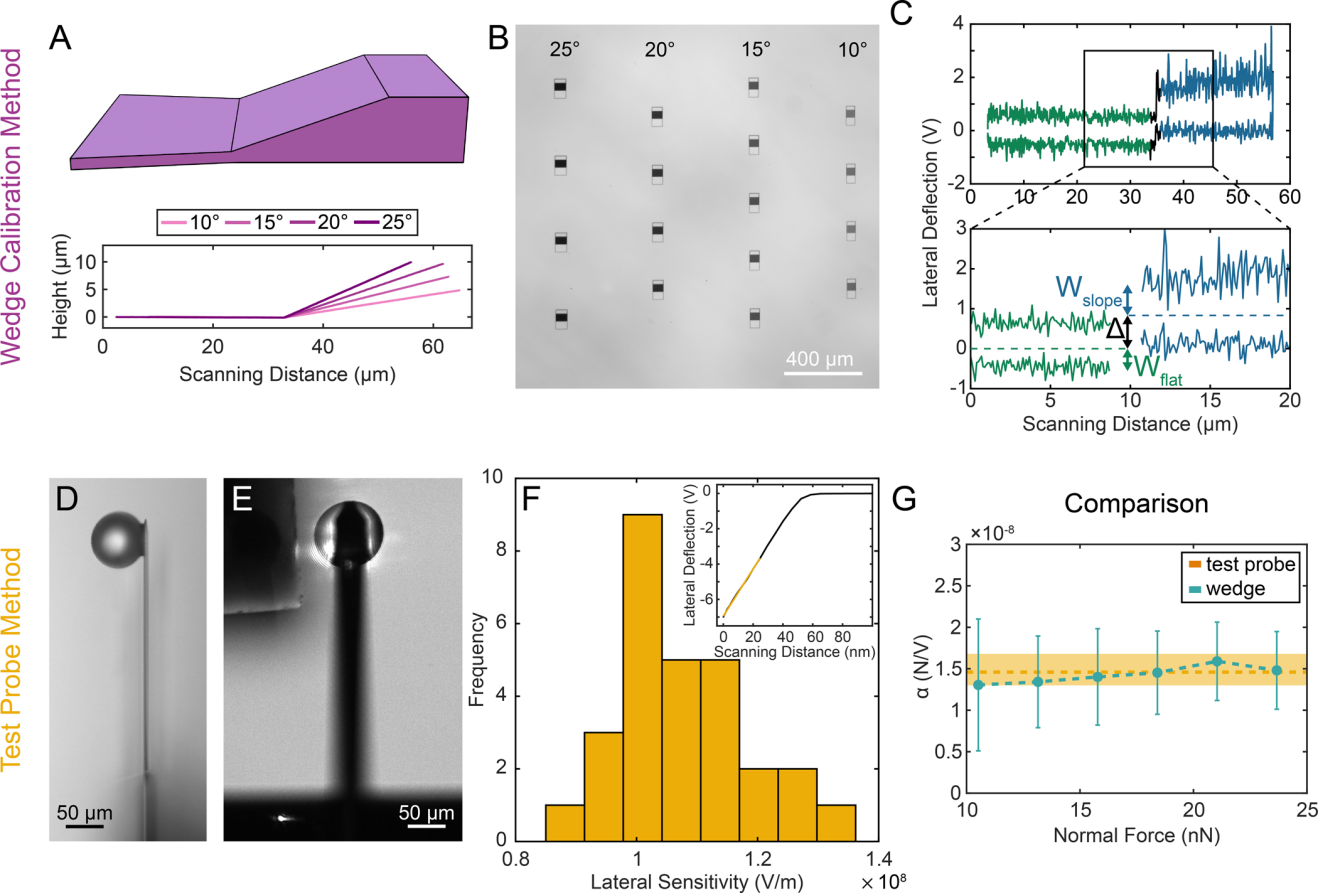
Comparison of two lateral-force calibration methods for the 3D-printed probes (wedge-calibration versus test-probe method). (A) 3D model of the wedges and corresponding height profiles, obtained from AFM linescans. (B) Optical micrograph of the wedges, fabricated using 3D 2PP-DLW. (C) Representative friction loop obtained on the wedge (top) and separated into the flat and sloped part (bottom). (D) Side-view of the large colloidal probe (silica, 90 μm) used for the test-probe method. (E) Top-view of the test probe against a sharp edge. (F) Histogram of repeated measurements of the lateral sensitivity. The inset depicts a representative deflection-vs-distance curve. (G) Comparison between the test probe-method and the wedge method in terms of sensitivity α.

The AFM cantilever is the force-sensing component of our methodology. According to Amontons’ law,^45^ the lateral force, *F*, scales linearly with the normal force, *L*, yielding the friction coefficient μ = *F*/*L*. Both force components are concurrently detected through the cantilever’s torsional and vertical deflection movements measured by a photodetector. A calibration procedure is thus required to relate the voltage response of the photodetector to a corresponding force value of the two separate deflections. Calibration of the normal-force component was conducted using the established Sader method,^46^ and the optical-lever sensitivity was obtained using the slope of the force-vs-distance curve obtained on a hard substrate within the contact regime. Many different lateral-force calibration methods have been developed over the years.^47^ Due to our unconventional probe design, we carried out a comparative analysis of two well-established ones, namely the wedge calibration method^38,48,49^ and the test-probe method.^39^ The comparison is shown in Figure 2. Originally developed by Ogletree et al.,^48^ the wedge calibration method was later refined^38,49^ to work for colloidal probes. The calibration factor was obtained by analyzing the cantilever deflections on two well-defined slopes. To accommodate the relatively large dimensions of our probe, we fabricated custom test substrates with wedge shapes using 3D 2PP-DLW (see the Experimental Section for more details). As shown in Figure 2A, wedges of different angles were designed and AFM height profiles confirmed the angles of the physical substrates. The correct torsion arm length was only provided when a particle was in the probe, therefore, to ensure a correct calibration, a particle was glued inside the holder before calibration. This step dictates that calibration is performed at the end of the free colloid experiments. Friction loops are recorded on all wedges in Figure 2B, at normal forces ranging from 10 to 80 nN. Figure 2C shows a representative loop, recorded on the 25°-slope, with *W* and Δ representing the half-width and the offset of the loop, respectively.^38^ The calibration factor α was directly obtained from the probe of interest in one step. The test-probe method, on the other hand, combines a contact-free torsional-spring-constant method^40^ with the measurement of the lateral-signal deflection sensitivity,^39^ obtained from a test probe of similar cantilever dimensions, but much larger colloid diameter. A representative test probe is shown in Figure 2D, where a 90 μm silica sphere was attached to a tipless AFM cantilever similar to that used for the free colloidal probe. The sensitivity was determined by loading the test probe against a vertical wall, as seen in Figure 2E, to obtain lateral-deflection vs scanning-distance plots. The histogram in Figure 2F shows the exceptional reproducibility observed over multiple lateral-sensitivity measurements. Finally, the calibration factor combines the torsional spring constant and the lateral sensitivity of the target cantilever, corrected for the different geometry. In Figure 2G, the two methods are compared, showing that they are in good agreement with each other. More details about the fabrication of the calibration wedges and the test probes can be found in the Experimental Section.

### 3.4 Driving Factors for Rolling Motion

The lateral force acting on the particle of a conventional colloidal probe depends on the applied normal force and the contact interactions with the substrate. In our case, when the particle is not fixed to the probe, the resulting motion and friction of the particle depend on the particle–substrate interaction as well as the holder–particle interaction. When the interaction between the holder and the particle is significantly higher than that between the particle and substrate, the traction force generated by the interaction with the substrate may not be sufficient to overcome the static friction between the particle and the holder. In such a scenario, the particle is stuck to the holder, and the measured friction force corresponds to the sliding friction of the particle with the substrate. If the torque resulting from the particle’s traction on the surface exceeds the reaction torque produced by static friction between the particle and the holder, rolling motion occurs. In this case, the measured friction results from a combination of particle–substrate rolling friction and particle-holder sliding friction and, in particular, a measure of the traction required to overcome sliding.

**Figure 3:**
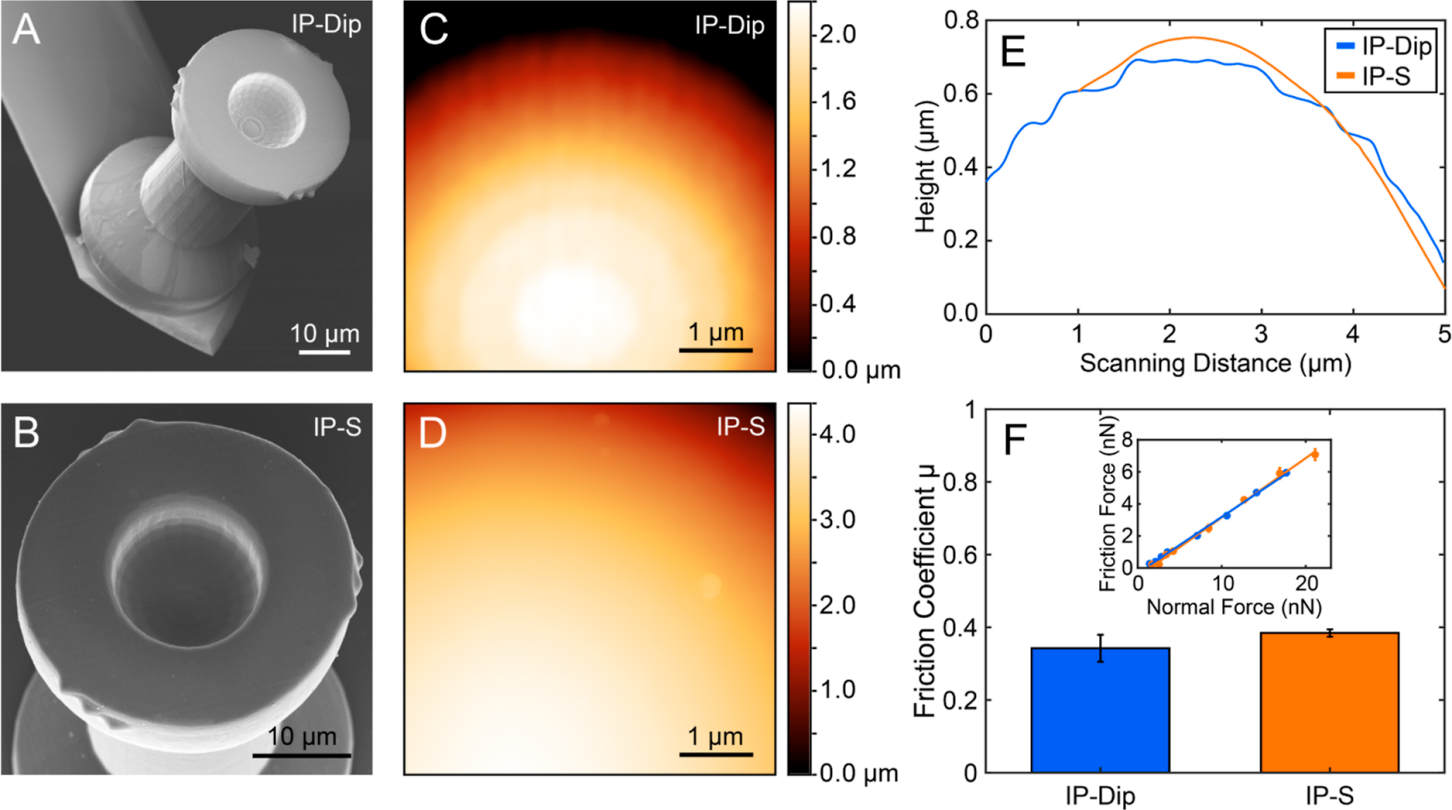
Effect of internal holder roughness. (A) SEM image of a holder, fabricated using the IP-Dip photoresin. (B) SEM image of a holder, fabricated using the IP-S photoresin. (C) AFM topography scan of a hemisphere representing a negative of the holder cavity from (A). (D) AFM topography scan of a hemisphere representing a negative of the holder cavity from (B). (E) Comparison of AFM height profiles, extracted from (C,D). (F) Comparison of the friction coefficient of a rough particle on a substrate with similar asperities, measured with holders fabricated with IP-Dip and IP-S, respectively. The inset shows a representative plot of friction force versus normal force.

The onset of rolling motion can be tuned by changing the surface properties in the cavity of the holder and hence the particle-holder contact interactions. Using two different commercially available resins to fabricate the holders, we investigated the effect of the internal roughness of the holder on the measured friction. With similar mechanical properties, IP-Dip (Young’s modulus = 1.3 GPa, NanoScribe GmbH, Germany) is optimized for highest-resolution printing and IP-S (Young’s modulus = 2.1 GPa, NanoScribe GmbH, Germany) for medium-sized features. When prints made from the two resins are compared, as seen in Figure 3A,B, it is clear that the holder made from IP-S (the lower resolution resin) has more rounded and smoother features than those of IP-Dip. Due to geometrical constraints, roughness measurements inside the holder were not feasible, so an exact negative of the hemisphere was fabricated in the same way as the holder itself. The AFM topography scan in Figure 3C,D clearly shows the difference in the roughness between holders made from the two resins. This becomes even more evident in Figure 3E, where a comparison of the two height profiles of the topographies highlights the steps in the print produced by IP-Dip.

To test if the roughness of the holder affects the measured friction, we compared holders made with both resins and performed friction experiments using a model system of rough, RB particles^35^ (silica, 12 μm microparticle decorated with 300 nm nanoparticle) on a rough substrate (silica, 100 nm nanoparticles). Details of particle and substrate fabrication methods can be found in the Experimental Section. The results show that the roughness of the holder has no effect on the measured friction coefficients, as seen in Figure 3F. Additionally, the motion of rough particles is almost identical and consists of almost pure rolling across the measured normal force range. Details can be found in Figure S3. Without adhesion, but with geometrical interlocking due to surface topography, intermittent rolling is observed at all applied normal forces. The contribution of rolling friction to the overall recorded lateral force is very low, considering that the sliding friction of a representative RB particle (12 μm microparticle with 300 nm nanoparticles) against a flat substrate made from the same resin as the holder (IP-S resin) is μ = 0.41 ± 0.13 (see Figure S4).

**Figure 4:**
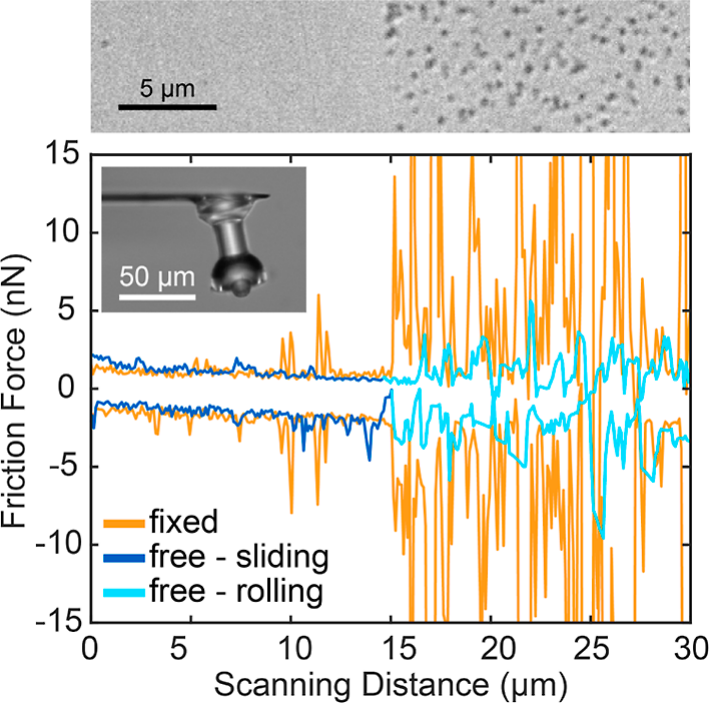
Friction loops of a fixed (orange) and a free (blue) RB particle, displaced across the patterned substrate at an applied normal force of 15 nN. The optical micrograph shows the change in roughness on the substrate (top). The inset shows an optical micrograph of the colloidal probe after converting it to a conventional fixed colloidal probe by gluing a RB particle inside the cavity of the holder.

The traction required for a particle to roll can originate from different phenomena, such as adhesion or gear-like interlocking of roughness on the particle and the substrate. For a clear demonstration of the onset of rolling, we displaced the same rough RB particles (silica, 12 μm microparticle decorated with 300 nm nanoparticles) at a scanning speed of 10 μm/s across a patterned substrate with a discontinuous roughness step, prepared according to the procedure described in the Experimental Section. The particle–substrate contact interactions change locally, therefore also changing the friction force and the motion, as seen in Figure 4. Within one 50 μm scan, the RB particle moves across the smooth part first, where the RB particle does not have enough traction to roll, according to the rotational analysis (see Figure S5). This is visualized in dark blue in Figure 4. Rolling motion is initiated whenever the shear stress at the particle–substrate contact overcomes the static friction at the particle holder contact. Based on the sliding friction of a RB particle against a flat substrate made from the holder material (see Figure S4), we estimate the necessary lateral force to initiate rolling here to be at least 5 nN, which is not reached. When the RB particle crosses the border to the rough part, the asperities interlock, creating traction that initiates rolling. However, the average measured friction remains almost identical with that of the smooth part despite the drastic change in roughness. The distinct spikes in the friction signal can be attributed to interlocking events of the asperities, but due to the possibility to roll, in the light-blue region in Figure 4, the friction remains relatively low, and clearly lower than in the case of the fixed particle. In Figure S6, we furthermore show that the fluctuations in lateral force are correlated to variations in the normal force, confirming that they originate from topography. This process is instantaneous and reproducible, clearly demonstrating that the motion state of a particle depends on the contact interactions with the substrate. It is important to note that the categorization into “sliding” and “rolling” is based on whether a rotation of ≥2° between frames occurred. The sampling rate of the force signal is around 3.5× higher than the acquisition rate of the fluorescence images, so some events in the force signals may not be picked up in the particle motion analysis. A fixed colloidal probe, obtained by gluing a similar RB particle inside the holder to restrict any rotation, experienced much larger friction forces on the rough substrate and pronounced stick–slip-like behavior, as seen in Figure 4 in orange. The spikes in the friction force of the fixed particle exceed the estimated threshold value of 5 nN to initiate rolling, as observed with the free RB particle (note: the friction loop of the fixed RB particle was obtained on the same sample as the free RB particle but not in the same location). The friction on the smooth surface obtained from a fixed RB particle is identical to the friction obtained from the probe with a free RB particle, since in both cases pure sliding is observed.

**Figure 5:**
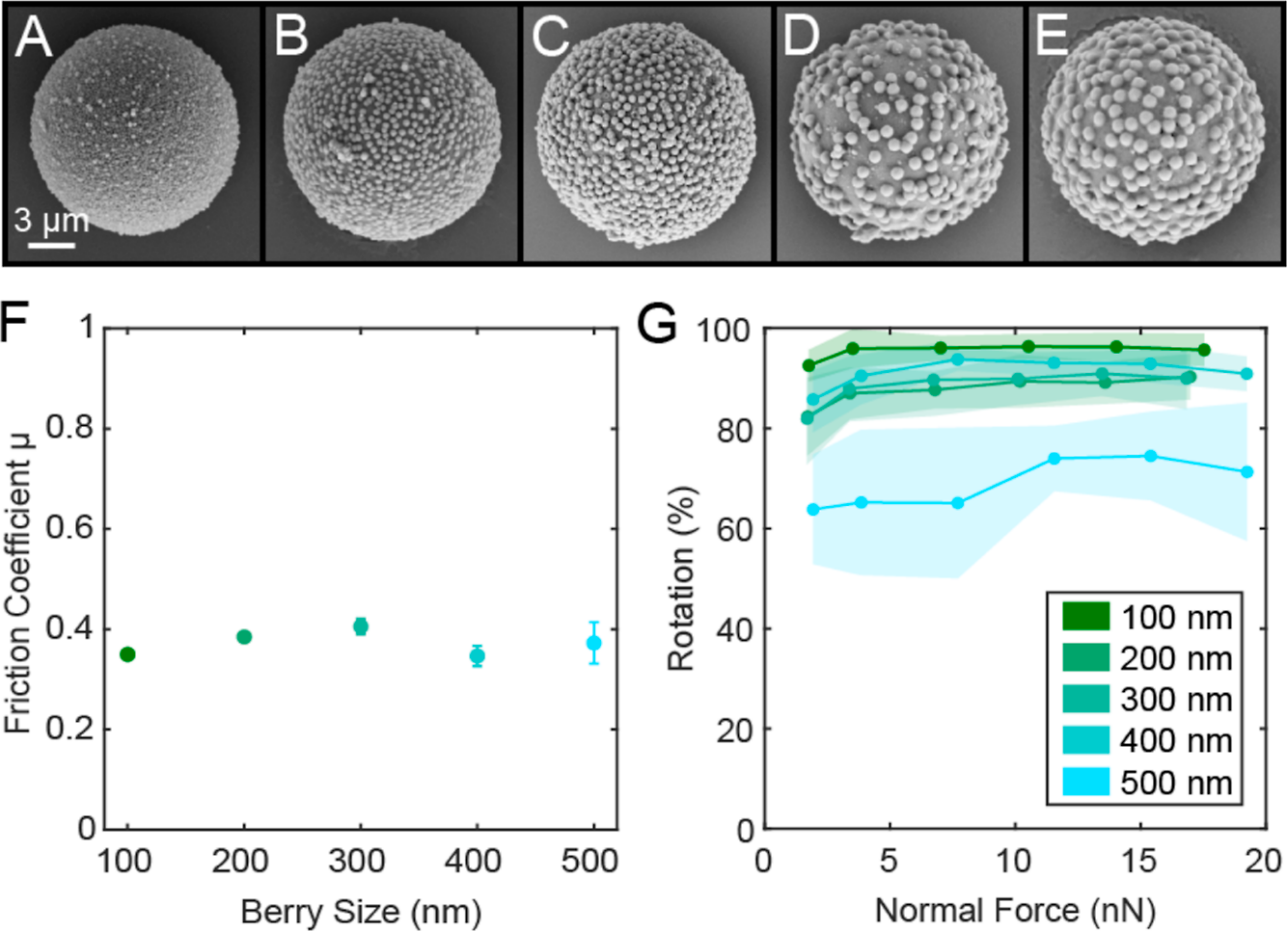
Raspberry (RB) particles (12 μm microparticle) with different roughness, expressed as nanoparticle diameter. (A) 100, (B) 200, (C) 300, (D) 400, and (E) 500 nm. (F) Effective friction coefficient μ of RB particles with different nanoparticle diameters (100–500 nm) on a rough substrate decorated with 100 nm nanoparticles. (G) Evolution of the rotation of the RB particles in (F), expressed as a percentage relative to pure rolling without slip, as a function of the applied normal force.

If the roughness of the substrate, over which a particle moves, plays a critical role in determining whether it rolls or slides, then the same goes for the surface properties of the particle, too. Due to the necessity of having fluorescent markers on the particle surface for rotational tracking, our particles have an inherent roughness, whose magnitude can be controlled via the addition of nanoparticles of different sizes. To study the effect of particle roughness on rolling/sliding friction, we synthesized particles using the same core microparticle diameter (12 μm), but varying the nanoparticle size between 100 and 500 nm, as seen in Figure 5A–E. The overall diameter of the smoothest and roughest particles does not change by more than 6%, and all particles were scanned on the same substrate using the same probe for the best comparison. The roughness of the substrate was matched to that of the smoothest particle by bonding 100 nm nanoparticles on a glass slide. The effective friction coefficient (Figure 5F), as well as the rotation coefficient (Figure 5G), remains constant for RB particles decorated with nanoparticles up to 400 nm. By measuring the amount of rotation, we observe that motion is close to pure rolling, indicating that strong interlocking occurs with little slip. Only RB particles decorated with 500 nm nanoparticles show less rolling, probably due to the increased difference in asperity size between the RB particles and the substrate, in turn leading to reduced interlocking. Nevertheless, the effective friction remains similar to that of particles with smaller nanoparticles, indicating that the contact interactions with the holder remain the same.

**Figure 6:**
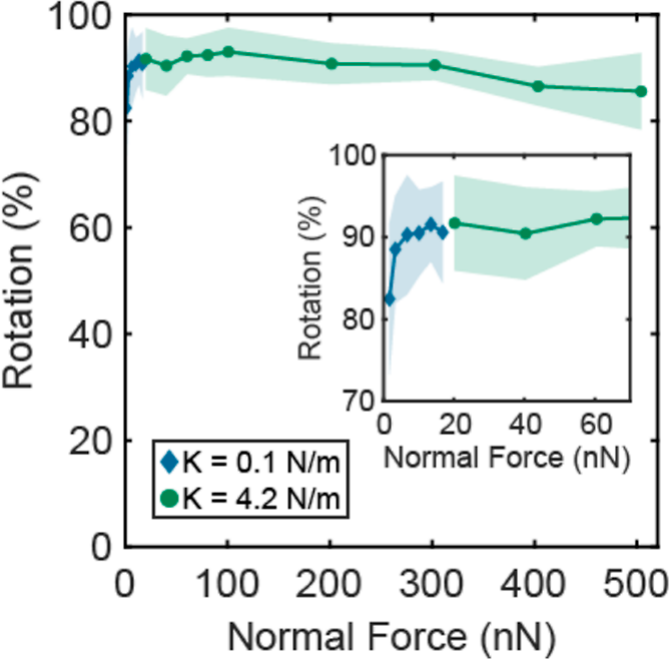
Rotation of raspberry (RB) particles (12 μm microparticle with 300 nm nanoparticles) on a rough substrate (100 nm nanoparticles) as a function of normal force over a broad range of normal forces applied by two cantilevers of different stiffness.

Finally, we investigated whether the observed rolling motion is affected by the applied normal force. To cover a wider range of applied normal forces, we used cantilevers of different normal stiffness of 0.1 and 4.2 N/m, to analyze the motion of rough RB particles (12 μm microparticles decorated with 300 nm nanoparticles) on a rough substrate (100 nm nanoparticles). As shown in Figure 6, we observed the saturation of the degree of rotation in the applied normal force region up to 500 nN and matching results using the two employed cantilevers. They also display a similar friction force, as seen in Figure S7. At low applied normal force, the rotation decreases, indicating that the particle does not fully engage with the substrate asperities to have the required traction to roll.

## 4 Conclusions

In this paper, we have described an innovative approach, building upon the foundation of lateral force microscopy with fixed colloidal probes, to extend the capabilities of LFM by enabling measurements of the friction of free particles moving over a surface. We established a reproducible workflow for the fabrication of LFM probes that can be applied to a broad range of particle sizes and materials and demonstrated the viability of two independent calibration methods routinely used for conventional colloidal probes. Furthermore, the effect of different holder materials was investigated, revealing that the internal roughness of the holder has a minimal impact. Experimental results from a rough RB particle, scanned over a substrate with discontinuous roughness, showcase instantaneous changes in friction and motion, indicating that rolling occurs when sufficient traction is provided by the surface roughness. A direct comparison between free and fixed particles reveals much lower effective friction for free particles, highlighting the impact of particle mobility on the energy dissipation by different contact forces. The highest traction is achieved when the roughness length scale on the substrate matches the size of the asperities on the particle, whereas a significant mismatch leads to more sliding. Using stiff cantilevers, we have also investigated contacts at applied normal forces up to 500 nN and observed that the rolling motion is consistently present under high-applied-normal-force conditions.

In conclusion, while conventional colloidal probe lateral force microscopy has firmly established itself as a powerful tool to investigate nanoscale friction, lubrication, and wear, our method opens up new possibilities that enable deeper insight into the intricate dynamics of sliding and rolling friction in nanoscale contacts. Gaining a more profound understanding of the nanomechanics of rolling contacts will, in fact, play a crucial role in the design of functional coating materials with engineered rolling friction. Moreover, it will help in elucidating further the role of interparticle contacts for a broad range of particle-based materials such as colloidal gels and particulate suspensions.

## Data Availability

All data needed to evaluate the conclusions in the paper are present in the paper and/or the Supporting Information. Additional data related to this paper may be requested from the authors upon reasonable request. The .stl-file of the 3D model of the holder can be found at https://doi.org/10.3929/ethz-b-000664345.

## Supporting Information Available

Synthesis and characterization of the raspberry particles used in this study, probe assembly procedure, data on averaged total rotation, friction force vs. normal force plot, data on angular displacements, data on representative lateral and vertical deflection signals, data on the friction force of a free raspberry particle, and 3D model of the holder (PDF)
