# Supplementary material for: Measuring Rolling Friction at the Nanoscale
Source: Langmuir. 2024 Mar 18;40(13):6750–60. doi: 10.1021/acs.langmuir.3c03499 (PMC10993404; doi:10.1021/acs.langmuir.3c03499)
Supplement: Supplementary file 1 — la3c03499_si_001.pdf [file la3c03499_si_001.pdf]

# Supporting Information: Measuring rolling friction at the nanoscale

Simon Scherrer, Shivaprakash N. Ramakrishna,* Vincent Niggel, Nicholas D. Spencer, and Lucio Isa*

*Department of Materials, ETH Zürich, 8093 Zürich, Switzerland*

E-mail: shivaprakash.ramakrishna@mat.ethz.ch; lucio.isa@mat.ethz.ch

## Supporting Information Available

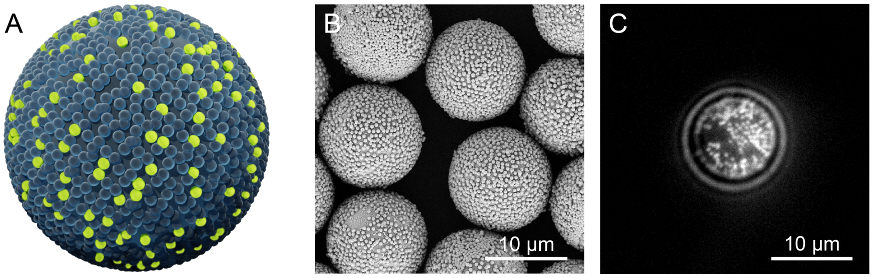


Figure S1: Raspberry (RB) particles used in this study. A) Representative schematic illustration of a RB particle comprising of a 12 $\mu$m microparticle core with defined surface roughness due to the surface-bound nanoparticles and fluorescent markers that allow for rotational tracking. B) SEM image of RB particles comprising of 12 $\mu$m microparticles decorated with 300 nm nanoparticles. C) Fluorescence microscopy image of a single RB particle comprising of a 12 $\mu$m microparticle decorated with 300 nm nanoparticles.

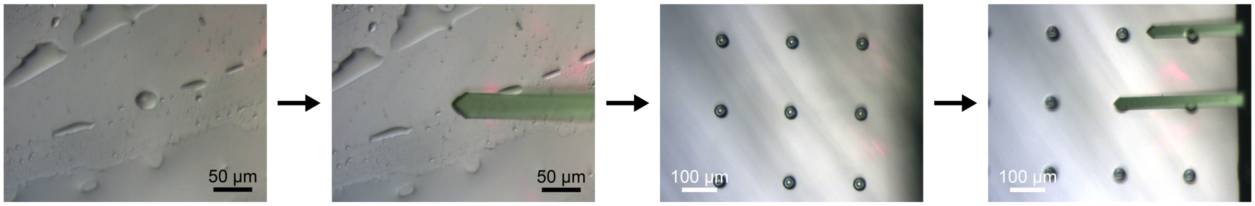


Figure S2: Probe assembly procedure. Small glue droplets are created by spreading UV adhesive with a micropipette tip before dipping a tipless AFM cantilever into one of the small drops. Finally, the cantilever tip is aligned with a holder, brought into contact, and cured using a UV light.

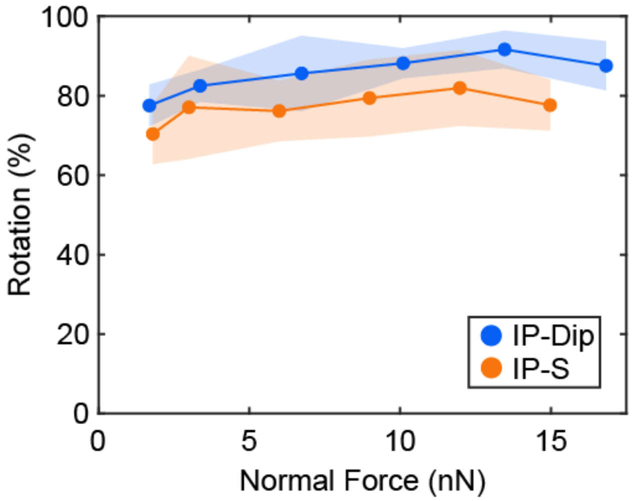


Figure S3: Averaged total rotation, represented as a percentage of pure rolling without slip, for similar RB particles (12 $\mu$m microparticle decorated with 300 nm nanoparticle) on the rough substrate (100 nm silica nanoparticles), measured with 2 holders made from different resins (IP-Dip and IP-S), as a function of the applied normal force.

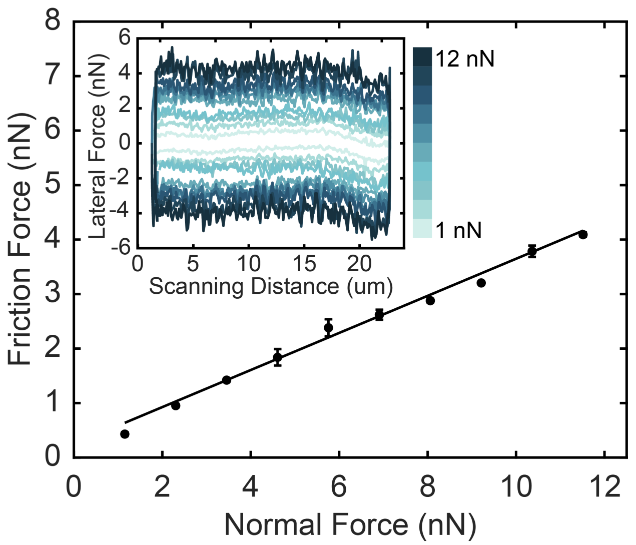


Figure S4: Friction force vs. normal force plot of a fixed RB particle (12 $\mu$m microparticle decorated with 300 nm nanoparticles) against a flat substrate made from the holder material (IP-S resin). The inset shows representative friction loops at normal forces ranging from 1 to 12 nN from which the friction force was calculated.

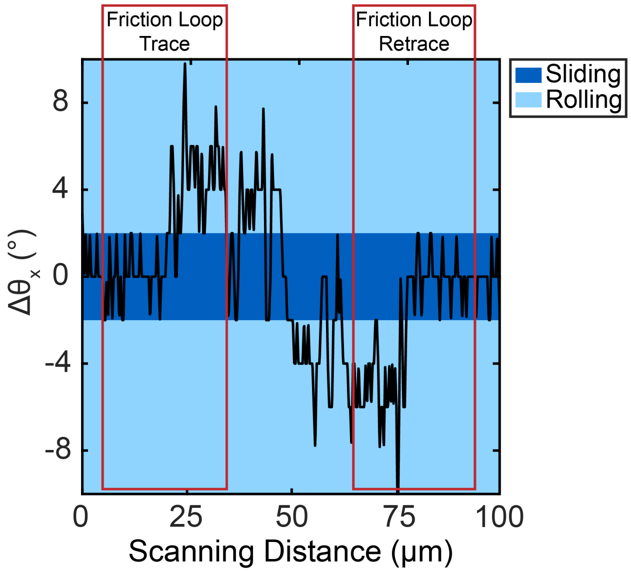


Figure S5: Angular displacements around the main rotation (x-)axis as a function of scanning distance for one full friction loop. The threshold value for rolling is set to 2°. The red box represents the section that is represented in the friction loop in Figure 4 of the main manuscript.

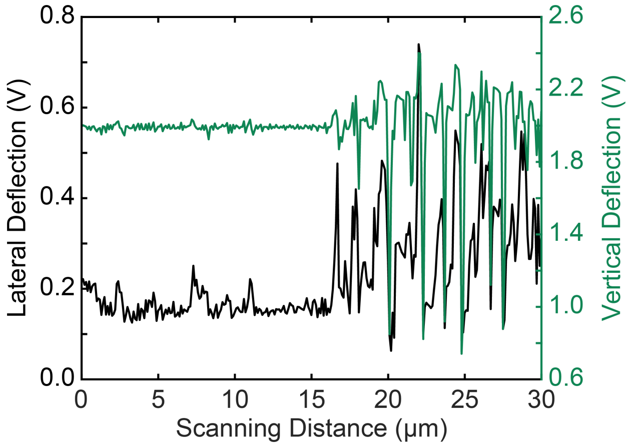


Figure S6: Representative lateral and vertical deflection signals of a free RB particle being displaced across a partially smooth (left) and partially rough (right) substrate. The deflection signals correspond to the blue friction loop of a free RB particle in Figure 4.

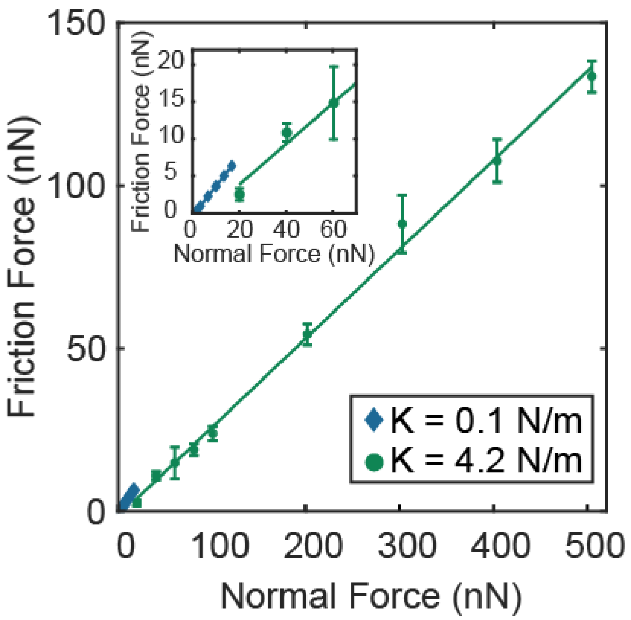


Figure S7: Friction force of a free raspberry particle (12 $\mu$m microparticle decorated with 300 nm nanoparticles on a rough substrate covered with 100 nm nanoparticles) as a function of applied normal force with two cantilevers of different stiffness.
